# A Thiazolidinedione Improves *In Vivo* Insulin Action on Skeletal Muscle Glycogen Synthase in Insulin-Resistant Monkeys

**DOI:** 10.1155/EDR.2000.195

**Published:** 2000

**Authors:** Heidi K. Ortmeyer, Noni L. Bodkin, Joseph Haney, Shinji Yoshioka, Hiroyoshi Horikoshi, Barbara C. Hansen

**Affiliations:** 1 Obesity and Diabetes Research Center Department of Physiology School of Medicine University of Maryland Baltimore MD 21201 USA; 2 Sankyo Company Ltd. Tokyo Japan

## Abstract

Thiazolidinediones (TZD) have been shown to have anti-diabetic effects including the ability to decrease fasting hyperglycemia and hyperinsulinemia, increase insulin-mediated glucose disposal rate (M) and decrease hepatic glucose production, but the mechanisms of action are not well established. To determine whether a TZD (R-102380, Sankyo Company Ltd., Tokyo, Japan) could improve insulin action on skeletal muscle glycogen synthase (GS), the rate-limiting enzyme in glycogen synthesis, 4 insulin-resistant obese monkeys were given I mg/kg/ day R-102380 p.o. for a 6-week period. Skeletal muscle GS activity and glucose 6-phosphate (G6P) content were compared between pre-dosing and dosing periods before and during the maximal insulin-stimulation of a euglycemic hyperinsulinemic clamp.

Compared to pre-dosing, insulin-stimulated GS activity and G6P content were increased by this TZD: GS independent activity (*p* = 0.02), GS total activity (*p* =  0.005), GS fractional activity (*p* = 0.06) and G6P content (*p* = 0.02). The change in GS activity induced by *in vivo* insulin (insulin-stimulated minus basal) was also increased by this TZD: GS independent activity (*p* = 0.03) and GS fractional activity (*p* = 0.04).

We conclude that the TZD R-102380 improves insulin action at the skeletal muscle in part by increasing the activity of glycogen synthase. This improvement in insulin sensitivity may be a key factor in the anti-diabetic effect of the thiazolidinedione class of agents.


*Int. Jnl. Experimental Diab. Res.*, Vol. 1, pp. 195–202
Reprints available directly from the publisher
Photocopying permitted by license only

© 2000 OPA (Overseas Publishers Association) N.V.
Published by license under
the Harwood Academic Publishers imprint,
part of The Gordon and Breach Publishing Group.
Printed in Malaysia.

### A Thiazolidinedione Improves *In Vivo* Insulin Action on Skeletal Muscle Glycogen Synthase in Insulin-resistant Monkeys

HEIDI K. ORTMEYER[a,*], NONI L. BODKIN[a], JOSEPH HANEY[a], SHINJI YOSHIOKA[b], HIROYOSHI HORIKOSHI[b] and BARBARA C. HANSEN[a]

[a]*Obesity and Diabetes Research Center, Department of Physiology, School of Medicine, University of Maryland, Baltimore, MD 21201;* [b]*Sankyo Company Ltd., Tokyo, Japan*

*(Received in final form 13 December 1999)*

**Thiazolidinediones (TZD) have been shown to have anti-diabetic effects including the ability to decrease fasting hyperglycemia and hyperinsulinemia, increase insulin-mediated glucose disposal rate (M) and decrease hepatic glucose production, but the mechanisms of action are not well established. To determine whether a TZD (R-102380, Sankyo Company Ltd., Tokyo, Japan) could improve insulin action on skeletal muscle glycogen synthase (GS), the rate-limiting enzyme in glycogen synthesis, 4 insulin-resistant obese monkeys were given 1 mg/kg/day R-102380 p.o. for a 6-week period. Skeletal muscle GS activity and glucose 6-phosphate (G6P) content were compared between pre-dosing and dosing periods before and during the maximal insulin-stimulation of a euglycemic hyperinsulinemic clamp.**

**Compared to pre-dosing, insulin-stimulated GS activity and G6P content were increased by this TZD: GS independent activity ($p = 0.02$), GS total activity ($p = 0.005$), GS fractional activity ($p = 0.06$) and G6P content ($p = 0.02$). The change in GS activity induced by *in vivo* insulin (insulin-stimulated minus basal) was also increased by this TZD: GS independent activity ($p = 0.03$) and GS fractional activity ($p = 0.04$).**

**We conclude that the TZD R-102380 improves insulin action at the skeletal muscle in part by increasing the activity of glycogen synthase. This improvement in insulin sensitivity may be a key factor in the anti-diabetic effect of the thiazolidinedione class of agents.**

*Keywords*: Thiazolidinedione; Glycogen synthase; Insulin action; Skeletal muscle; Rhesus monkey

### INTRODUCTION

Thiazolidinediones have been shown to reduce fasting and postprandial hyperglycemia and to increase insulin-mediated glucose disposal rate in type 2 diabetic humans,[13] to decrease fasting and postprandial concentrations of insulin and triglycerides in obese insulin-resistant rhesus monkeys,[10] to decrease hepatic glucose production in diabetic mice[6] and in normal rats,[11] and to inhibit glucagon-stimulated glycogen breakdown and gluconeogenesis in normal rat hepatocytes.[2] The mechanism of the insulin sensitizing effect of the thiazolidine-

*Corresponding author. Tel.: 410-706-3904, Fax: 410-706-7540, e-mail: hortmeye@umaryland.edu diones is not known, although there is evidence that they may act by regulating the expression of genes involved in glucose and lipid metabolism in adipose tissue and in skeletal muscle through binding with the peroxisome proliferator-activated receptor-$\gamma$.[7, 21]

During a euglycemic hyperinsulinemic clamp the skeletal muscle is the primary site for glucose disposal, and glycogen storage is the primary pathway of this glucose disposal. Insulin activation of skeletal muscle glycogen synthase, the rate-limiting enzyme in glycogenesis, is strongly related to insulin-mediated glucose disposal rate in humans and in rhesus monkeys.[3, 16] Insulin-mediated skeletal muscle glycogen synthase activity has been shown to be reduced in insulin-resistant states.[3, 16] Although several studies have examined the effects of thiazolidinediones on glycogen synthase activity *in vitro*,[1, 5, 20] the effect of thiazolidinediones on *in vivo* insulin action on skeletal muscle glycogen synthase has not been previously reported.

The purpose of the present study was to determine whether a thiazolidinedione could improve *in vivo* insulin action during a euglycemic hyperinsulinemic clamp by promoting an increase in skeletal muscle glycogen synthase activity in insulin-resistant monkeys.

### MATERIALS AND METHODS

This chronic study of thiazolidinedione treatment was carried out in four obese male insulin-resistant rhesus monkeys (Macaca mulatta) that had never received insulin treatment. Baseline pre-dosing measurements included 2 hour post-vehicle plasma glucose and insulin determinations and a euglycemic hyperinsulinemic clamp with tissue biopsies obtained prior to the onset of the clamp (basal), and during maximal insulin-stimulation. The pre-dosing baseline measurements were reported previously in a study which included the determination of *in vivo* insulin action on liver, subcutaneous and omental adipose tissue and on skeletal muscle glycogen synthase activity.[14] Monkeys were then treated for 6 weeks with R-102380 (Sankyo Company Ltd., Tokyo, Japan) (1 mg/kg/day p.o.). At the end of this 6-week period, the 2-hour post drug plasma glucose and insulin concentrations were determined and the euglycemic hyperinsulinemic clamp with biopsies was repeated.

The euglycemic hyperinsulinemic clamp was performed as previously described in these animals.[14] The glycogen synthase assay and the glucose 6-phosphate assay were performed as described previously.[16, 18] The glucose 6-phosphate Ka of glycogen synthase was determined as described previously.[19] Briefly, glycogen synthase activity was measured in the absence and in the presence of the following glucose 6-phosphate concentrations: 0.10, 0.25, 0.50, 0.75, 1, 2.5, 5, 7.5 and 10 mmol/l. The glucose 6-phosphate Ka of glycogen synthase (the apparent affinity of glycogen synthase for glucose 6-phosphate) was then determined by

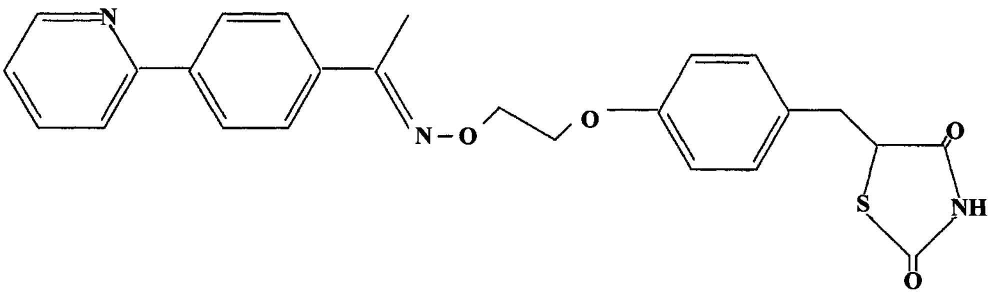

FIGURE 1 Structure of the thiazoladinedione R-102380.

plotting the glucose 6-phosphate concentration against the glucose 6-phosphate concentration divided by velocity ($s/v$ against $s$ plot).[9] Total protein was measured in the supernatant using the Bradford method.[4]

Data are expressed as mean ± SE. Differences in basal and insulin-stimulated values were determined using a paired $t$-test.

The structure of the thiazolidinedione R-102380 is shown in Figure 1.

### RESULTS

The characteristics of the individual rhesus monkeys before and at the end of the six-week TZD treatment period are shown in Table I. For these 4 adult insulin-resistant monkeys, considered as a group, administration of the TZD R-102380, at a dose of 1 mg/kg/day for six weeks, did not produce significant changes in body weight, plasma glucose, plasma insulin, or whole-body insulin-mediated glucose disposal rate. Nevertheless, it appears that the TZD positively affected tissue insulin sensitivity in every monkey, and that the individual monkeys strongly differed in the ways in which the TZD affected them. We suspect these individual differences were dependent upon how far along the progression toward overt diabetes each monkey was at the time of study.

The monkey (J-8) that was hyperglycemic prior to TZD treatment showed an improvement in plasma glucose and an increase in plasma insulin levels commensurate with an improvement in the plasma insulin curve by reversing the course followed during the natural progression toward overt type 2 diabetes.[8] This monkey, reasonably considered the most diabetic of the group, showed the greatest improvement in insulin sensitivity. A second monkey (A-7), which was also declining toward diabetes prior to TZD treatment, also showed a similar increase in insulin sensitivity. Two normoglycemic insulin-resistant monkeys (R-8 and S-8) showed major declines in plasma insulin levels, in accord with their position at an earlier phase in the progression toward diabetes (at this early stage, improvement in hyperinsuliemia is shown by a lowering of plasma insulin concentrations); thus, they showed improved beta-cell function, although these two monkeys showed no measurable improvement in insulin sensitivity, at least as assessed by the euglycemic hyperinsulinemic clamp.

Basal skeletal muscle glycogen synthase activity for each individual monkey is presented in Figure 2, with independent, total, and fractional activity shown for the pre-dosing period and during dosing. For the group as a whole, the basal glycogen synthase activities were not significantly effected by R-102380 (pre-R-102380

TABLE I Characteristics of rhesus monkeys before and during R-102380

| | Age | Body fat | Body weight (kg) | | Glucose* (mmol/l) | | Insulin* (pmol/l) | | M† (mg/kg FFM/min) | |
|---|---|---|---|---|---|---|---|---|---|---|
| ID | (Yr) | (%) | Pre | Post | Pre | Post | Pre | Post | Pre | Post |
| A-7 | 17 | 45.1 | 16.6 | 16.3 | 4.5 | 4.2 | 1464 | 1566 | 5.33 | 7.25 |
| J-8 | 16 | 26.4 | 11.3 | 10.7 | 11.5 | 8.9 | 258 | 306 | 6.58 | 8.79 |
| R-8 | 8 | 29.9 | 22.1 | 22.4 | 6.1 | 4.2 | 3636 | 1602 | 6.49 | 5.13 |
| S-8 | 8 | 26.8 | 15.8 | 16.1 | 2.9 | 3.3 | 1692 | 558 | 5.25 | 4.67 |

*Plasma glucose and insulin determinations were made during the pre-treatment period 2 hours following administration of vehicle and during dosing period 2 hours after the monkey received the same vehicle containing 1 mg/kg R-102380. † whole-body insulin-mediated glucose disposal rate.

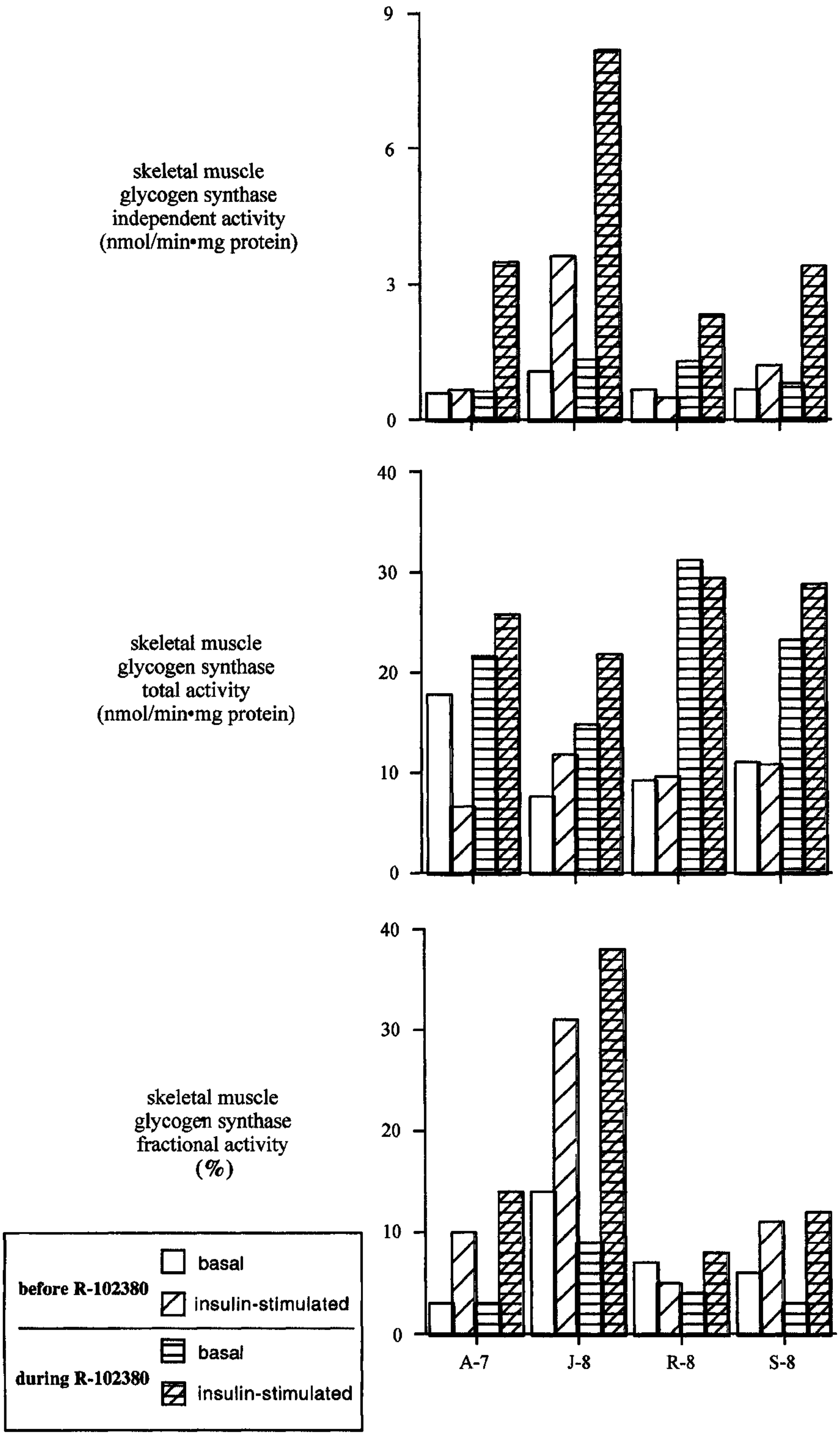

FIGURE 2 Basal and insulin-stimulated skeletal muscle glycogen synthase activities before and after 6 weeks of treatment with R-102380 in 4 obese insulin-resistant monkeys. The mean insulin-stimulated activities before *vs.* during R-102380 were significantly different for independent ($p = 0.02$) and for total ($p = 0.005$) activity. The mean insulin effect (insulin-stimulated minus basal) before *vs.* during R-102380 was significantly different for independent ($p = 0.03$) and for fractional ($p = 0.04$) activity.

*vs.* during R-102380: independent activity, $0.73 \pm 0.12$ *vs.* $1.01 \pm 0.17$ nmol/min·mg protein, $p = 0.11$; total activity, $11.29 \pm 2.22$ *vs.* $22.60 \pm 3.34$ nmol/min·mg protein, $p = 0.07$; fractional activity, $7.5 \pm 2.3$ *vs.* $4.8 \pm 1.4\%$, $p = 0.08$). All individual monkeys showed an increase in both basal glycogen synthase independent activity and basal glycogen synthase total activity; however, the individual monkeys varied in the magnitudes of their responses, thus, preventing significance by the usual paired *t* tests. The small but consistent increases in basal glycogen synthase independent activity were accompanied by much larger increases in total glycogen synthase activity, and thus, the glycogen synthase fractional activity (proportion of glycogen synthase in the active form [independent] relative to total glycogen synthase) was reduced during treatment relative to pre-treatment.

Under insulin stimulation during a euglycemic hyperinsulinemic clamp, skeletal muscle glycogen synthase activity was also examined, as shown in Figure 2. For the group as a whole, the TZD significantly increased insulin-stimulated glycogen synthase activity: insulin-stimulated glycogen synthase independent activity was increased 3 fold ($1.5 \pm 0.7$ *vs.* $4.4 \pm 1.3$ nmol/min/mg protein, $p = 0.02$), and total glycogen synthase activity was also increased nearly 3 fold ($9.6 \pm 1.1$ *vs.* $26.3 \pm 1.7$ nmol/min/mg protein, $p = 0.005$). Glycogen synthase fractional activity under insulin stimulation tended to be increased ($14 \pm 6$ *vs.* $18 \pm 7\%$, $p = 0.06$). As for basal glycogen synthase activity, every individual monkey showed an increase in insulin-stimulated glycogen synthase activity – independent, total, and fractional activity, but the magnitude of the increment induced by the TZD varied across animals.

R-102389 significantly enhanced insulin action (insulin-stimulated minus basal) on glycogen synthase independent ($0.76 \pm 0.62$ *vs.* $3.35 \pm 1.23$ nmol/min/mg protein, $p = 0.03$) and fractional activities ($6.8 \pm 3.9$ *vs.* $13.3 \pm 5.5\%$, $p = 0.04$), but had no significant effect on the change in glycogen synthase total activity in response to insulin ($-1.67 \pm 3.31$ *vs.* $3.74 \pm 2.00$, $p = 0.24$). The magnitude of the TZD effect on independent glycogen synthase activity was much greater (about 3 fold) under insulin stimulation than under the basal condition. The greatest absolute increase in glycogen synthase independent activity and in glycogen synthase fractional activity with R-102380 treatment was observed in the most diabetic monkey (J-8), the monkey that also showed the greatest increase in whole-body insulin sensitivity as measured by the euglycemic hyperinsulinemic clamp.

Basal glucose 6-phosphate content was not significantly effected by R-102380 ($0.47 \pm 0.17$ *vs.* $0.73 \pm 0.15$ nmol/mg dry weight, $p = 0.13$). Glucose 6-phosphate content under insulin-stimulated conditions was significantly higher during dosing ($0.43 \pm 0.08$ nmol/mg dry weight) than before dosing ($0.27 \pm 0.09$ nmol/mg dry weight) ($p = 0.02$) (Fig. 3).

Neither the basal, insulin-stimulated nor insulin effect on the glucose 6-phosphate Ka of glycogen synthase were significantly effected by R-102380 (basal, $2.34 \pm 0.94$ *vs.* $2.78 \pm 0.85$ mmol/l, $p = 0.13$; insulin-stimulated, $1.15 \pm 0.45$ *vs.* $0.73 \pm 0.19$ mmol/l, $p = 0.28$; insulin-stimulated minus basal, $-1.19 \pm 1.02$ *vs.* $-2.05 \pm 0.73$ mmol/l, $p = 0.17$) (Fig. 3). Basal *vs.* insulin-stimulated glucose 6-phosphate Ka of glycogen synthase during dosing approached significance ($p = 0.07$).

The total basal protein concentrations before R-102380 and during R-102380 administration were $0.89 \pm 0.12$ *vs.* $1.23 \pm 0.09$ mg/ml, respectively ($p = 0.09$). The total protein concentrations under insulin-stimulated conditions before R-102380 and during R-102380 administration were $0.94 \pm 0.04$ *vs.* $1.16 \pm 0.10$ mg/ml, respectively ($p = 0.11$).

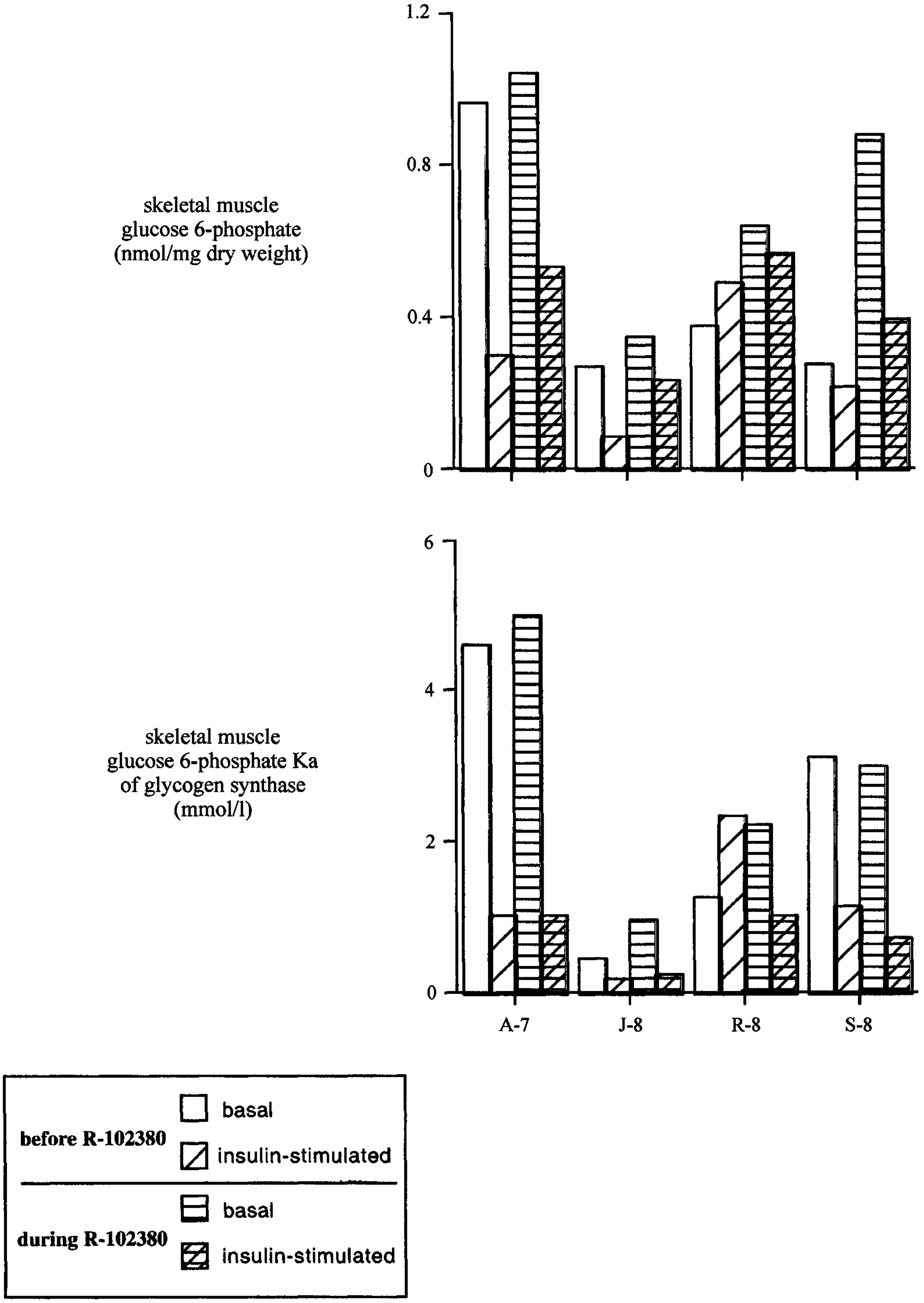

FIGURE 3 Basal and insulin-stimulated skeletal muscle glucose 6-phosphate content and glucose 6-phosphate Ka of glycogen synthase before and after 6 weeks of treatment with R-102380. The mean before *vs.* during R-102380 was significantly different for insulin-stimulated glucose 6-phosphate content ($p = 0.02$).

### DISCUSSION

Insulin activation of skeletal muscle glycogen synthase by dephosphorylation is a good measure of insulin sensitivity. In normal rhesus monkeys, *in vivo* insulin during a euglycemic hyperinsulinemic clamp caused a significant increase in the independent activity of skeletal muscle glycogen synthase without changing total glycogen synthase activity.[16] This resulted in a significant increase in the fractional activity of the enzyme. In addition, *in vivo* insulin during a euglycemic hyperinsulinemic clamp resulted in a significant decrease in the glucose 6-phosphate Ka of glycogen synthase in normal (ad libitum-fed) rhesus monkeys,[17] which is consistent with an increase in dephosphorylation of the enzyme. In obese hyperinsulinemic and type 2 diabetic monkeys, the effect of insulin to increase glycogen synthase independent activity was significantly less than that of the normal monkeys.[16] The effect of insulin to covalently activate muscle glycogen synthase was strongly related to whole-body insulin-mediated glucose disposal rate in these monkeys.[16]

In the present study of insulin-resistant rhesus monkeys, 6 weeks of treatment with a thiazolidinedione (R-102380) resulted in a significant increase in insulin sensitivity as measured by an increase in insulin action on skeletal muscle glycogen synthase independent and fractional activity. A similar finding has been reported on the effect of the thiazolidinedione AD-5075 to increase *in vitro* insulin stimulation of rat adipose tissue glycogen synthase.[1] In that study, AD-5075 was added to adipose tissue *in vitro* and the tissue cultured for 5 hours. AD-5075 caused a significant increase in basal independent glycogen synthase activity as well as in *in vitro* insulin-stimulated independent glycogen synthase activity. Total glycogen synthase activity was not significantly affected by AD-5075, either in the basal or insulin-stimulated conditions.[1]

In another study of *in vitro* thiazolidinedione effect on glycogen synthase, troglitazone was added to skeletal muscle cultures of type 2 diabetic subjects.[20] Troglitazone was shown to significantly increase glycogen synthase fractional activity under *in vitro* insulin stimulation compared to basal activity. Although the effect of troglitazone on total glycogen synthase activity was not reported in that study, chronic troglitazone did not effect either mRNA or protein concentrations of glycogen synthase.[20]

In the present study, 6 weeks of treatment with R-102380 did not cause a significant change in the glucose 6-phosphate Ka of glycogen synthase, either during basal or insulin-stimulated conditions. This is in contrast to an *in vitro* study in which the thiazolidinedione CS-045 was added to cultured liver cells.[5] In that study, the addition of CS-045 caused a 3 fold decrease in the glucose 6-phosphate Ka of glycogen synthase; however, CS-045 also caused a significant decrease in total glycogen synthase activity in the Hep G2 cells.[5] In addition, CS-045 did not increase glycogen synthase independent activity above the increase seen in the presence of *in vitro* insulin in either Hep G2 or BC3H-1 cells.[5]

In the present study, the most profound effect of R-102380 was an increase in skeletal muscle glycogen synthase total activity during the euglycemic hyperinsulinemic clamp. This suggests that administration of R-102380 increased glycogen synthase protein expression in the presence of *in vivo* insulin. *In vivo* insulin during a euglycemic hyperinsulinemic clamp has not been shown to increase skeletal muscle glycogen synthase total activity in normal, prediabetic or diabetic monkeys or in chronically calorie-restricted monkeys,[14–16] neither has it been shown to increase protein expression or mRNA expression in humans.[12, 22, 23]

In summary, the thiazolidinedione R-102380 improves *in vivo* insulin action on skeletal muscle glycogen synthase in insulin-resistant rhesus monkeys; this may be an important mechanism by which thiazolidinediones improve insulin sensitivity. The effect of R-102380

to increase glycogen synthase total activity during a euglycemic hyperinsulinemic clamp warrants the future investigation of the effect of thiazolidinediones on skeletal muscle glycogen synthase protein and mRNA expression.

### Acknowledgements

The authors would like to thank the following people for their excellent technical support of this study: Theresa Alexander, Wallace Evans, Jr., Karen Brocklehurst, Alana Dunevant and Teerin Meckmongkol.

### References

[1] Berger, J., Biswas, C., Hayes, N., Ventre, J., Wu, M. and Doebber, T. W. (1996). An antidiabetic thiazolidinedione potentiates insulin stimulation of glycogen synthase in rat adipose tissue. *Endocrinology*, **137**, 1984-1990.

[2] Blackmore, P. F., McPherson, R. K. and Stevenson, R. W. (1993). Actions of the novel antidiabetic agent englitazone in rat hepatocytes. *Metabolism*, **42**, 1583-1587.

[3] Bogardus, C., Lillioja, S., Stone, K. and Mott, D. (1984). Correlation between muscle glycogen synthase activity and *in vivo* insulin action in man. *J. Clin. Invest.*, **73**, 1185-1190.

[4] Bradford, M. M. (1976). A rapid and sensitive method for the quantitation of microgram quantities of protein utilizing the principle of protein-dye binding. *Anal. Biochem.*, **72**, 248-254.

[5] Ciaraldi, T. P., Gilmore, A., Olefsky, J. M., Goldberg, M. and Heidenreich, K. A. (1990). *In vitro* studies on the action of CS-045, a new antidiabetic agent. *Metabolism*, **39**, 1056-1062.

[6] Fujiwara, T., Okuno, A., Yoshioka, S. and Horikoshi, H. (1995). Suppression of hepatic gluconeogenesis in long-term Troglitazone treated diabetic KK and C57BL/KsJ-db/db mice. *Metabolism*, **44**, 486-490.

[7] Hallakou, S., Doaré, L., Foufelle, F., Kergoat, M., Guerre-Millo, M., Berthault, M. -F., Dugail, I., Morin, J., Auwerx, J. and Ferré, P. (1997). Pioglitazone induces *in vivo* adipocyte differentiation in the obese Zucker *fa/fa* rat. *Diabetes*, **46**, 1393-1399.

[8] Hansen, B. C. and Bodkin, N. L. (1986). Heterogeneity of insulin responses: phases in the continuum leading to non-insulin-dependent diabetes mellitus. *Diabetologia*, **29**, 713-719.

[9] Henderson, P., Statistical analysis of enzyme kinetic data. In: *Enzyme Assays: A Practical Approach*, edited by Eisenthal, R. and Danson, M. Oxford: IRL press, 1993, pp. 277-316.

[10] Kemnitz, J., Elson, D., Roecker, E., Baum, S., Bergman, R. and Meglasson, M. (1994). Pioglitazone increases insulin sensitivity, reduces blood glucose, insulin, and lipid levels, and lowers blood pressure in obese, insulin-resistant rhesus monkeys. *Diabetes*, **43**, 204-211.

[11] Lee, M. K. and Olefsky, J. M. (1995). Acute effects of troglitazone on *in vivo* insulin action in normal rats. *Metabolism*, **44**, 1166-1169.

[12] Mandarino, L. J., Printz, R. L., Cusi, K. A., Kinchington, P., O'Doherty, R. M., Osawa, H., Sewell, C., Consoli, A., Granner, D. K. and DeFronzo, R. A. (1995). Regulation of hexokinase II and glycogen synthase mRNA, protein, and activity in human muscle. *Am. J. Physiol.*, **269**, E701-E708.

[13] Mimura, K., Umeda, F., Hiramatsu, S., Taniguchi, S., Ono, Y., Nakashima, N., Kobayashi, K., Masakado, M., Sako, Y. and Nawata, H. (1994). Effects of a new oral hypoglycemic agent (CS-045) on metabolic abnormalaties and insulin resistance in type 2 diabetes. *Diabetic Medicine*, **11**, 685-691.

[14] Ortmeyer, H. K. and Bodkin, N. L. (1998). Lack of defect in insulin action on hepatic glycogen synthase and phosphorylase in insulin-resistant monkeys. *Am. J. Physiol.*, **274**, G1005-G1010.

[15] Ortmeyer, H. K., Bodkin, N. L. and Hansen, B. C. (1994). Chronic caloric restriction alters glycogen metabolism in rhesus monkeys. *Obes. Res.*, **2**, 549-555.

[16] Ortmeyer, H. K., Bodkin, N. L. and Hansen, B. C. (1993). Insulin-mediated glycogen synthase activity in muscle of spontaneously insulin-resistant and diabetic rhesus monkeys. *Am. J. Physiol.*, **265**, R552-R558.

[17] Ortmeyer, H. K., Bodkin, N. L. and Hansen, B. C. (1999). Paradoxical phosphorylation of skeletal muscle glycogen synthase by *in vivo* insulin in very lean young adult rhesus monkeys. *Annals New York Academy of Sciences*, **892**, 247-260.

[18] Ortmeyer, H. K., Bodkin, N. L. and Hansen, B. C. (1994). Relationship of skeletal muscle glucose-6-phosphate to glucose disposal rate and glycogen synthase activity in insulin-resistant and non-insulin-dependent diabetic rhesus monkeys. *Diabetologia*, **37**, 127-133.

[19] Ortmeyer, H. K., Huang, L., Larner, J. and Hansen, B. C. (1998). Insulin unexpectedly increases the glucose 6-phosphate Ka of skeletal muscle glycogen synthase in calorie-restricted monkeys. *J. Basic Clin. Physiol. Pharmacol.*, **9**, 309-323.

[20] Park, K. S., Ciaraldi, T. P., Abrams-Carter, L., Mudaliar, S., Nikoulina, S. E. and Henry, R. R. (1998). Troglitazone regulation of glucose metabolism in human skeletal muscle cultures from obese type II diabetic subjects. *J. Clin. Endocrinol. Metab.*, **83**, 1636-1643.

[21] Park, K. S., Ciaraldi, T. P., Lindgren, K., Abrams-Carter, L., Mudaliar, S., Nikoulina, S. E., Tufari, S. R., Veerkamp, J. H., Vidal-Puig, A. and Henry, R. R. (1998). Troglitazone effects on gene expression in human skeletal muscle of type II diabetes involve up-regulation of peroxisome proliferator-activated receptor-gamma. *J. Clin. Endocrinol. Metab.*, **83**, 2830-2835.

[22] Schalin-Jäntti, C., Laurila, E., Löfman, M. and Groop, L. C. (1995). Determinants of insulin-stimulated skeletal muscle glycogen metabolism in man. *European Journal of Clinical Investigation*, **25**, 693-698.

[23] Vestergaard, H., Bjorbaek, C., Andersen, P. H., Bak, J. F. and Pedersen, O. (1991). Impaired expression of glycogen synthase mRNA in skeletal muscle of NIDDM patients. *Diabetes*, **40**, 1740-1745.

